# Encoding-related brain activity and accelerated forgetting in transient epileptic amnesia

**DOI:** 10.1016/j.cortex.2018.04.015

**Published:** 2019-01

**Authors:** Kathryn E. Atherton, Nicola Filippini, Adam Z.J. Zeman, Anna C. Nobre, Christopher R. Butler

**Affiliations:** a Nuffield Department of Clinical Neurosciences, University of Oxford, John Radcliffe Hospital, Oxford, UK; b Cognitive & Behavioural Neurology, University of Exeter Medical School, Exeter, UK; c Department of Experimental Psychology and Oxford Centre for Human Brain Activity, Wellcome Centre for Integrative Neuroimaging, Department of Psychiatry, University of Oxford, Oxford, UK

**Keywords:** Accelerated forgetting, Memory, Epilepsy, fMRI, Transient epileptic amnesia, ALF, accelerated long-term forgetting, MTL, medial temporal lobe, ROI, region of interest, SEM, standard error of the mean, TEA, transient epileptic amnesia

## Abstract

The accelerated forgetting of newly learned information is common amongst patients with epilepsy and, in particular, in the syndrome of transient epileptic amnesia (TEA). However, the neural mechanisms underlying accelerated forgetting are poorly understood. It has been hypothesised that interictal epileptiform activity during longer retention intervals disrupts normally established memory traces. Here, we tested a distinct hypothesis–that accelerated forgetting relates to the abnormal encoding of memories. We studied a group of 15 patients with TEA together with matched, healthy control subjects. Despite normal performance on standard anterograde memory tasks, patients showed accelerated forgetting of a word list over one week. We used a subsequent memory paradigm to compare encoding-related brain activity in patients and controls. Participants studied a series of visually presented scenes whilst undergoing functional MRI scanning. Recognition memory for these scenes was then probed outside the scanner after delays of 45 min and of 4 days. Patients showed poorer memory for the scenes compared with controls. In the patients but not the controls, subsequently forgotten stimuli were associated with reduced hippocampal activation at encoding. Furthermore, patients demonstrated reduced deactivation of posteromedial cortex regions upon viewing subsequently remembered stimuli as compared to subsequently forgotten ones. These data suggest that abnormal encoding-related activity in key memory areas of the brain contributes to accelerated forgetting in TEA. We propose that abnormally encoded memory traces may be particularly vulnerable to interference from subsequently encountered material and hence be forgotten more rapidly. Our results shed light on the mechanisms underlying memory impairment in epilepsy, and offer support to the proposal that accelerated forgetting may be a useful marker of subtle dysfunction in memory-related brain systems.

## 1 Introduction

Transient epileptic amnesia (TEA) is a syndrome of temporal lobe epilepsy in which recurrent, brief amnestic seizures, which often occur upon waking, are typically associated with persistent interictal memory difficulties (Butler et al., 2007, Zeman et al., 1998). Even once the seizures have been successfully treated with anticonvulsant medication, patients with TEA can often learn information at a normal rate but forget it excessively rapidly thereafter, particularly over intervals longer than the standard 30-min test delay, the phenomenon of ‘accelerated long-term forgetting’ (ALF) (e.g., Butler and Zeman, 2008, Hoefeijzers et al., 2014, Muhlert et al., 2010).

The scientific study of accelerated forgetting is important for both clinical and theoretical reasons. The phenomenon is now recognised to occur amongst patients with other forms of epilepsy, often goes undetected on standard clinical neuropsychology assessments and causes considerable disability (Elliott et al., 2014, Fitzgerald et al., 2013a, Fitzgerald et al., 2013). Furthermore, recent work indicates that ALF might be a sensitive marker of very early, even pre-symptomatic Alzheimer's disease (Walsh et al., 2014, Weston et al., 2018, Zimmermann & Butler, 2018). Investigation of the underlying mechanisms of accelerated forgetting will provide insight into healthy memory processes and could help in the development of novel diagnostic and therapeutic tools. Although at first sight the phenomenon might appear likely to result from an isolated impairment of memory consolidation, here we explore whether abnormalities in memory encoding may play a significant role.

Accelerated forgetting in TEA appears to be specific to declarative memories (Muhlert et al., 2010). The hippocampus and neighbouring structures in the medial temporal lobes (MTL) are critical for this type of memory (e.g., Squire, 1992, Squire and Zola-Morgan, 1991). While TEA patients' clinical MRI scans are typically normal, the patients show subtle hippocampal atrophy at the group level (Butler et al., 2009, Butler et al., 2013), and the seizure focus is thought to reside in the MTL (e.g., Zeman & Butler, 2010).

A major role of the hippocampus and surrounding structures is thought to be the formation of representations that allow us to discriminate between similar experiences (e.g., Bussey et al., 2005, Graham et al., 2010, Lee et al., 2005, Marr, 1971, O'Reilly and McClelland, 1994, Treves and Rolls, 1994). If the hippocampus were not functioning properly, memories with overlapping components could severely interfere with one another.

One possible explanation for accelerated forgetting is that patients have mild hippocampal dysfunction, resulting in the production of substandard memory representations that are vulnerable to interference. If this were the case, one might expect hippocampal activity to be abnormal at the stage of encoding, even when behavioural performance is initially normal as is often the case in TEA (e.g., Atherton et al., 2014, Butler and Zeman, 2008, Hoefeijzers et al., 2013); behavioural deficits would only appear once interference had occurred. The current study uses functional magnetic resonance imaging to test the hypothesis that TEA patients have abnormal brain activity at the stage of encoding that relates to their subsequent memory performance.

Functionally relevant differences in memory encoding have commonly been studied using the subsequent-memory paradigm (e.g., Brewer et al., 1998, Paller et al., 1987; Wagner et al., 1998), in which the brain activity associated with viewing subsequently remembered stimuli is contrasted with that associated with subsequently forgotten stimuli. In these studies, successful encoding has often been associated with activity in MTL memory structures, including the hippocampus, and with prefrontal regions (especially the inferior frontal gyrus), the dorsal posterior parietal cortex, and the fusiform cortex (e.g., Spaniol et al., 2009, Uncapher and Wagner, 2009). Furthermore, successful encoding is typically associated with deactivation of the posteromedial cortex, including the posterior cingulate and precuneus (Daselaar et al., 2004, Huijbers et al., 2012, Otten and Rugg, 2001). These regions are strongly connected with the MTL and are thought to play a major role in hippocampus-dependent memory networks (Buckner et al., 2008, Sperling et al., 2010, Spreng et al., 2009, Wang et al., 2010).

Some studies have examined encoding-related brain activity in patient populations. For instance, research in temporal lobe epilepsy has found hypoactivity in the affected MTL (e.g., Bonnici et al., 2013, Das et al., 2009, Powell et al., 2007, Richardson et al., 2003, Voets et al., 2009, Voets et al., 2014) and, in some cases, abnormal recruitment of other brain regions (e.g., Powell et al., 2007, Richardson et al., 2003, Sidhu et al., 2013). Other studies have found the development of Alzheimer's disease to be associated with decreased hippocampal activity (Sperling et al., 2010) and a concomitant loss of normal deactivation of the posteromedial cortex (Celone et al., 2006, Miller et al., 2008, Pihlajamaki et al., 2008, Pihlajamaki et al., 2010, Sperling et al., 2010) during encoding. The present study is the first to examine brain activity during encoding in TEA patients with accelerated forgetting. These patients are unusual in that they typically have no gross brain damage and their interictal memory deficit often only becomes apparent over extended delays (Butler et al., 2007). TEA patients with accelerated forgetting therefore present a novel opportunity to investigate the relationship between neural processing at the stage of encoding and subsequent memory performance.

Our fMRI study design involved two subsequent memory tests – one shortly after encoding and one four days later. This design has the advantage of providing an opportunity to investigate the neural correlates of successful encoding of longer-term versus shorter-term long-term memories. The majority of subsequent-memory experiments to date have used only a single memory probe. The few that have used multiple probes over time, have typically found subsequent memory durability to be related to activity in the MTL or posterior cingulate cortex (e.g., Carr et al., 2010, Ritchey et al., 2008, Sneve et al., 2015, Uncapher and Rugg, 2005, Wagner et al., 2016).

We hypothesised that TEA patients who describe symptoms of accelerated forgetting would show brain activity abnormalities at the stage of encoding in the hippocampus or surrounding MTL that predict their subsequent memory performance.

## 2 Materials and methods

The study received ethical approval from the Scotland A Research Ethics Committee and written informed consent was obtained from all participants according to the Declaration of Helsinki.

### 2.1 Participants

Fifteen patients and 15 control participants took part. The patients met diagnostic criteria for TEA (Zeman & Butler, 2010) and described symptoms of accelerated forgetting.

The groups did not differ in terms of age, IQ or performance on a standard anterograde verbal memory test (see Table 1). Scores on this anterograde memory test were missing from one patient as he did not complete the task correctly. While no participants scored within the ‘abnormal’ range on either component of the Hospital Anxiety and Depression Scale, there was a significant group difference on the depression component of this scale. Therefore, depression scores were used as a covariate in our analyses. All patients were on anticonvulsant therapy and, in all cases but one, had been free of seizures for at least six months prior to testing. No patients reported seizures during the experiment. The control participants did not suffer from any psychiatric or central nervous system disorders.

**Table 1:** **Participant information.** Means with SEMs in brackets. The groups did not differ significantly in terms of age, IQ or the anxiety component of the HAD scale (*p* > .05). However, there was a group difference on the depression component of the HAD scale (t_(23.97)_ = −2.46, *p* = .021).

	TEA Patients	Controls
N	15	15
Gender	3 female	5 females
Age	67.73 (±1.63)	63.50 (±1.44)
*IQ and standard memory tests*		
Predicted WAIS^a^ verbal IQ from NART^b^ errors	115.33 (±2.36)	118.93 (±1.64)
WASI^c^ similarities raw score (max 48)	39.20 (±.92)	39.40 (±.97)
WASI matrix reasoning raw score (max 42)	26.53 (±.92)	24.27 (±1.48)
WMS-III^d^ Logical Memory immediate recall	14.20 (±3.83)	17.1 (±4.22)
WMS-III Logical Memory delayed recall	11.21 (±5.44)	14.60 (±4.53)
*Anxiety and depression scores*		
Hospital Anxiety and Depression Scale (HADS)^e^ anxiety (max 21)	6.07 (±.62)	4.73 (±.61)
Hospital Anxiety and Depression Scale (HADS) depression (max 21)	4.73 (±.80)	2.40 (±.51)*

∗*p*<0.001

a WAIS = Wechsler Abbreviated Intelligence Scale (Wechsler, 1955).

b NART (H. Nelson & Willison, 1991; H. E. Nelson, 1982).

c WASI = Wechsler Abbreviated Scale of Intelligence (Wechsler, 1999).

d WMS-III = Wechsler Memory Scale-III (Wechsler, 1997).

e HADS (Zigmond & Snaith, 1983).

### 2.2 Experimental design

#### 2.2.1 Main task

The experiment was designed and presented using Presentation software (Neurobehavioral systems, Albany, CA).

Each participant viewed a series of stimuli in the scanner. The stimuli were full colour digital images (768 × 512 pixels) derived from photographs of real-life scenes. The majority was taken from a stimulus set used by Voets et al. (2009) and Filippini et al. (2009).

The experiment used a mixed design. The stimuli were presented in alternating blocks of novel and repeated scenes. Each block lasted 13.5 sec and contained ten picture presentations. In the ‘repeated’ blocks, the same two images were presented repeatedly in a pseudorandom order. The same two images were used in every repeated block. In the ‘novel’ blocks, all scenes were unique, and shown only once in the experiment. To compare brain activity linked to subsequently remembered and forgotten items, the data analyses were conducted using an event-related approach based on the stimuli within the novel blocks.

Fifty percent of the scene stimuli were dominated by a picture of one or more animals. No animals appeared in the other images. The participant's task was to indicate, with a forced-choice button press whether or not the picture contained an animal. Participants were also instructed to memorise the stimuli.

The fMRI experiment was divided into two runs of sixteen blocks (eight novel, eight repeated), one immediately after the other, to minimise movement artefacts.

**Fig. 1:**
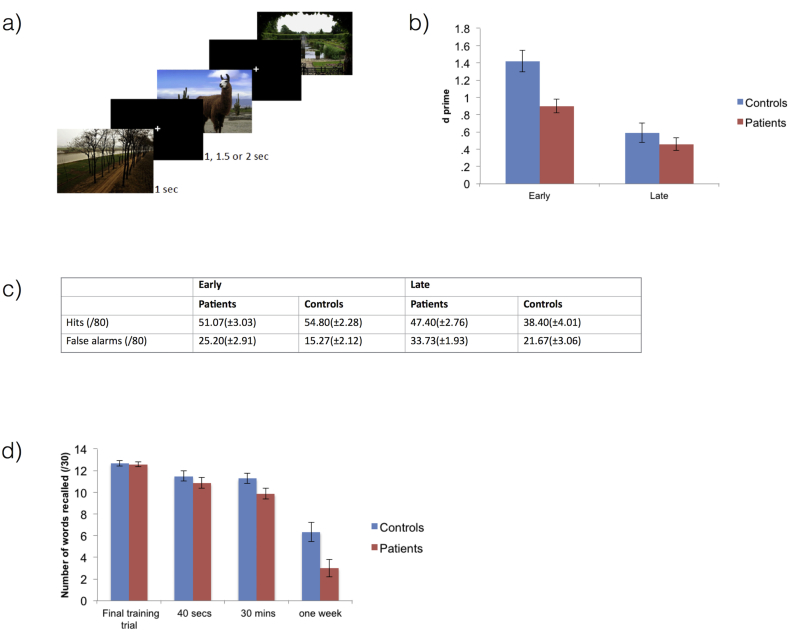
**Task and behavioural results.** (a) Three example stimuli from a novel block of the study task performed during the fMRI scan. Each image was presented for one second. There was an interval of varying duration (1, 1.5 or 2 s) between stimuli, during which a fixation cross was presented. The participant's task was to indicate, using an MR-compatible button box, whether each image contained an animal. The participant was also instructed to memorise the stimuli. (b) D′ in the Early and Late recognition memory tests of the fMRI task. The patients had a significantly lower d’ than the controls on the Early test, but not on the Late test. (c) The raw number of hits and false alarms in the Early and Late recognition tests. (d) Performance on the word-list recall task (RAVLT). The patients performed significantly more poorly than the controls on the 1-week test and the 30-min test, but not on the final training trial or the 40-s test. The percentage forgotten between the 30-min test and the 1-week test was significantly greater in the patients than the controls. The error bars represent SEMs.

In novel blocks, a total of 160 unique pictures were presented during the scan. Each picture was presented for one second (Fig. 1a). The intervening interstimulus interval contained only a fixation cross, and had a varying duration (1, 1.5 or 2 sec).

The first recognition test (*Early*) was performed soon after the participant left the scanner, approximately 45 min after the encoding task. This test was performed in a separate room from the scanner, on a laptop computer. No feedback was given. This was a test for half of the novel pictures that were presented in the scanner (80/160). The participant was shown 160 pictures, half of which were foils. For each image, the participant was asked to indicate whether the picture was old or new. The picture remained on the screen until a response was made. A fixation cross was presented for one second between stimuli.

Four days later, the participant completed another recognition test at home (the *Late* test), where they viewed the stimuli on a computer screen and gave responses for each image verbally over the telephone. The internet was used to deliver the images to the participant's computer on the day of testing. In the event that the participant did not have access to a computer (three cases), he/she was provided with a digital photoframe, preloaded with the test images. During the Late test, the participant was tested on the other half of the pictures that were presented during the scan, with the same number of novel foils (160 images in total).

The identity of the stimuli presented in the Early and Late tests was counterbalanced across participants; the targets used in the Early test for half of the participants were used in the Late test for the other half of the participants, and vice versa. The order of stimulus presentation within the Early and Late tests was randomised. The order in which the stimuli were presented in the scanner was also counterbalanced across participants; half of the participants viewed the blocks of novel and repeated pictures in one order, and the other half viewed the blocks in the reverse order.

#### 2.2.2 RAVLT word-list task

Participants were also tested on an adapted version of the Rey Auditory Verbal Learning Test (RAVLT, Schmidt, 1996). This is the test on which accelerated forgetting over the longer term (accelerated long-term forgetting, ALF) has most commonly been demonstrated in TEA patients. Participants were read a list of fifteen nouns aloud. The participant then attempted to recall as many of the words as possible in any order. If fewer than 12/15 were recalled, the list was read aloud again before they had a second attempt. This process was repeated until the participant reached the criterion of 12/15 words. The participant was then distracted for approximately 40 sec (during which time they were asked to count back from 100 in 3 sec) before being asked to recall as many of the words from the list as they could. Recall was probed again after 30 min, and then again after one week (over the telephone). One of the patients was not available to take the RAVLT, and one was excluded from analyses because he admitted to rehearsing the word-list during the intervening week. The interval between the MRI scanning session and the RAVLT learning session was not significantly different in patients and controls (−.92 months ± 2.98 and 2.73 months ± 1.63, *p* = .59).

### 2.3 Data acquisition

Imaging data were acquired using a 3 Tesla scanner (SIEMENS MAGNETOM Verio syngo MR B17) and a 32-channel head coil. Functional data were acquired using a gradient echo EPI (echo-planar imaging) sequence (TE: 30 msec, TR: 2410 msec, flip angle: 90°, field of view (FOV): 192 mm, phase encoding: anterior-posterior, GRAPPA Factor = 2, slices per volume: 44, slice thickness: 3 mm, voxel size: 3 × 3 × 3 mm). Two dummy scans from the beginning of each run were discarded, to allow for T1 saturation. Each of the two runs lasted 8 min and 21 sec.

High resolution structural images were acquired using a T1-weighted MEMPRAGE sequence (van der Kouwe, Benner, Salat, & Fischl, 2008) (slice orientation: sagittal, TR: 2530 msec, 4 TEs: 1.69 msec, 3.55 msec, 5.41 msec and 7.27 msec (the average image was used), flip angle: 7°, field of view: 256 mm, slice thickness: 1 mm, voxel size: 1 × 1 × 1 mm, acquisition time: 6 min and 3 sec).

### 2.4 Statistical analysis

#### 2.4.1 Behavioural analyses

Sensitivity (d’) was calculated for each recognition test of the main task as: Z (hit rate) – Z (false alarm rate). An ANCOVA was performed with d’ as the dependent variable, time (two levels: Early and Late) as the within-subjects factor, group as the between-subjects factor and depression score on the HAD scale as a nuisance covariate.

The data from the adapted RAVLT were analysed using an ANCOVA with number of words recalled as the dependent variable, time (four levels: final training trial; 40 sec; 30 min; and one week) as the within-subjects factor, group as the between-subjects factor and depression score on the HAD scale as a nuisance covariate. Long-term forgetting on this task was defined as the percentage of words forgotten between the 30-min test and the 1-week test and was compared in the two groups using a one-way ANCOVA with depression score on the HAD scale as a nuisance covariate.

Performance on the main task (Early and Late memory test d’) and the adapted RAVLT (percentage forgotten between the 30-min test and the 1-week test) was compared using partial correlations, with depression score on the HAD scale as a nuisance covariate.

#### 2.4.2 Imaging data

Imaging data were analysed using FSL (FMRIB's Software Library, Jenkinson, Beckmann, Behrens, Woolrich, & Smith, 2012) version 6.00. The structural images were reoriented and brain extracted using fsl_anat. Functional data were processed and analysed using FEAT (FMRIB's Expert Analysis Tool, part of FSL). Preprocessing included: brain extraction using BET (Brain Extraction Tool, Smith, 2002); motion correction with MCFLIRT (motion correction using FMRIB's Linear Image Registration tool, Jenkinson, Bannister, Brady, & Smith, 2002); B0 unwarping (which was carried out using BBR (Boundary-Based Registration, Greve & Fischl, 2009), and for which the fieldmap image was processed using fsl_prepare_fieldmap and the magnitude fieldmap image was skull stripped using BET, with option —B to reduce image bias); spatial smoothing (FWHM: 8 mm); and high pass temporal filtering (high pass filter cutoff: 132 sec for the block analysis and 90 sec for the event-related analyses).

The data were analysed using a General Linear Model, and temporal autocorrelation correction was achieved using FILM (FMRIB's Improve Linear Model, Woolrich, Ripley, Brady, & Smith, 2001) prewhitening. We coded each novel stimulus according to whether it was correctly identified as old (remembered) or incorrectly labelled as new (forgotten) in the subsequent Early and Late tests. The stimuli presented during repeated blocks were coded separately. In our main analysis, there were three experimental explanatory variables (EVs) at the first level: Remembered; Forgotten; and Repeated. In each case, the EV was generated using timing information from the Presentation logfiles to produce a square wave model of neural activity, which was then convolved with a double-gamma HRF (hemodynamic response function). For each experimental EV, a temporal derivative was added (to allow for differences in slice acquisition times, and slight variability in the timing of the explanatory variable stimulation and the HRF delay), and temporal filtering was used. Additional confound EVs (generated from the preprocessed data) were included in the analysis: fsl_motion_outliers was used to identify volumes corrupted by substantial motion; and the mean time courses from an ROI in the cerebrospinal fluid (CSF) of the anterior lateral ventricle and an ROI in the white matter of the dorsal posterior frontal lobe were used to account for physiological noise (Leech, Braga, & Sharp, 2012). These ROIs were 3 mm radius spheres centred on the MNI coordinates 2 10 8 and -26 -22 28, respectively, before registration to native space using nearest neighbour interpolation.

In a second analysis, in which the Early and Late tests were compared, there were five experimental EVs at the first level: Early remembered; Early forgotten; Late remembered; Late forgotten; Repeated. One participant (a control) was excluded from this analysis because he had no Late remembered trials in one of his fMRI runs.

Contrasts of the parameter estimates for the experimental explanatory variables (COPEs) were used in second-level analyses. The two runs from each participant were combined using a second-level fixed-effects analysis.

At the third level, mixed-effects voxel-wise group analyses were conducted. Unless stated otherwise, third level z-statistic images were thresholded using clusters identified by z > 2.3 and a corrected cluster significance threshold of *p* = .05 (Worsley, 2001). To ensure that any group differences in brain activity could not be accounted for by group differences in brain structure or depression scores, we added covariates for grey matter and HAD scale depression score into our third level Feat analyses. The FSL tool feat_gm_prepare was used to generate a grey matter density map from each subject's structural data; these maps can be used to produce voxelwise explanatory variables (e.g., Filippini et al., 2009, Hafkemeijer et al., 2013, Peraza et al., 2014, Westlye et al., 2011), making it possible to eliminate the confound of anatomical differences.

For each covariate, we demeaned across all participants and then generated two separate explanatory variables – one for the controls (with zero values for the patients) and one for the patients (with zero values for the controls). Having separable EVs allows FEAT to treat the two groups of participants as different, with different variances, increasing sensitivity to group differences. Our contrasts of interest had zero values for all of the nuisance explanatory variables.

Within FEAT, FLIRT_BBR was used to register each participant's functional data to his/her high-resolution structural image and FNIRT (Andersson, Jenkinson, & Smith, 2010) (with 12 degrees of freedom for the linear component, and a warp resolution of 10 mm) was used to register each participant's structural image to standard space (MNI-152 template).

The hippocampus was of particular interest in this study, as this brain area is implicated in both memory encoding and TEA. For region-of-interest (ROI) analyses, left and right hippocampus masks were taken from the Harvard–Oxford subcortical atlas, thresholded and binarised such that all voxels with an intensity value below 50 were excluded from the mask and all other voxels took a value of 1, and then used to constrain the FEAT analyses.

Featquery was used to probe certain results: a binary mask of the region of interest was created and then transformed into the native space of each participant. Featquery was then used to calculate the mean percent signal change for the relevant contrasts within the mask for each participant. Where relevant, statistical analyses were then performed on these data and considered significant when *p* < .05 (as was also the case for behavioural analyses).

## 3 Results

### 3.1 Behaviour

#### 3.1.1 Main task

Fig. 1b displays d’ for the patients and controls in the Early and Late memory tests. Fig. 1c displays the raw number of hits and false alarms. An ANCOVA with d’ as the dependent variable was conducted to test for effects of time (two levels: Early and Late) as the within-subjects factor, group as the between-subjects factor, and depression score on the HAD scale as a nuisance covariate. A main effect of group [F_(1,27)_ = 6.61, *p* = .016] showed patients performing more poorly (estimated marginal mean (EMM) and standard error of the mean (SEM): .66 ± .095) than the controls (EMM ± SEM: 1.02 ± .095). A main effect of time [F_(1,27)_ = 33.16, *p* < .001] showed performance declining between the Early test (EMM ±SEM: 1.16 ± .074) and the Late test (EMM ± SEM: .52 ± .066). An interaction between time and group [F_(1,27)_ = 10.89, *p* = .003] revealed patients performing significantly more poorly than controls on the Early test (adjusted means ± SEM: .87 ± .11 and 1.45 ± .11, *p* = .002) but not on the Late test (adjusted means ± SEM: .45 ± .098 and .59 ± .098, *p* = .32). Both controls (*p* < .001) and patients (*p* < .001) showed a significant performance decline with time. A univariate ANCOVA, with percentage change in d’ between 40 min and four days as the dependant variable and depression score on the HAD scale as a nuisance covariate showed no significant group difference between patients and controls (adjusted means ± SEM: −46.3 ± 31.7% and −62.4 ± 24.9%, *p* = .221). At four days, patients in particular were approaching floor level, with both hit (.59) and false alarm (.42) rates not far from chance (.50), and 8/15 patients having a d’ less than .5.

Patients' poorer performance in the Early memory test was driven by a higher false alarm rate in patients compared with controls (adjusted means ± SEM: .33 ± .032 and .17 ± .032, *p* = .003) rather than lower hit rate (adjusted means ± SEM: .65 ± .035 and .68 ± .035, *p* = .620). The same pattern was observed at the Late memory test with patients showing a higher false alarm rate (adjusted means ± SEM: .43 ± .033 and .26 ± .033, *p* = .001) but no difference in hit rate (adjusted means: ±SEM:0.60 ± .046 and .47 ± .046, *p* = .060).

#### 3.1.2 RAVLT word-list task

Both the training and testing of the subsidiary word-list task occurred outside the scanner. The patients and controls did not differ in the number of trials to reach criterion (4.46 ± .79 and 4.33 ± 1.19, respectively, *p* = .93). Recall performance is displayed in Fig. 1d.

An ANCOVA with words recalled as the dependent variable tested the effects of time (four levels: final training trial; 40 sec; 30 min; and one week) as the within-subjects factor, group as the between-subjects factor and depression score on the HAD scale as a nuisance covariate. A main effect of group showed patients performing more poorly on the task than controls [EMMs ± SEMs: 8.82 ± .41 and 10.64 ± .38, F_(1, 25)_ = 9.30, *p* = .005]. Also observed were a main effect of time [F_(1.74, 43.50)_ = 46.44, *p* < .001] and an interaction between time and group [F_(1.74, 43.50)_ = 5.87, *p* = .008]. Post-hoc *t*-tests showed that the groups did not perform differently on the final training trial (adjusted means ±SEMs: 12.46 ± .26 and 12.73 ± .24, *p* = .47) or the 40-sec test (adjusted means ± SEMs: 10.70 ± .52 and 11.59 ± .48, *p* = .25), but that patients performed more poorly than controls after 30 min (adjusted means ±SEMs: 9.63 ± .53 and 11.45 ± .49, *p* = .026) and one week (adjusted means ±SEMs: 2.47 ± .92 and 6.79 ± .84, *p* = .003). The percentage forgotten between the 30-min test and the 1-week test was greater in patients than controls [adjusted means ± SEMs: 74.50 ± 7.65% and 41.42 ± 7.05%, F_(1,25)_ = 8.91, *p* = .046], as revealed by a targeted subsidiary univariate ANCOVA with depression score on the HAD scale as a nuisance covariate.

Across all participants, controlling for group and depression score on the HAD scale, there was a strong correlation between percentage forgotten on the RAVLT between the 30-min and the 1-week tests and d’ on the picture recognition task at the Early memory test (partial r = −.592, *p* = .008) and a marginal correlation at the Late memory test (partial r = −.381, *p* = .055).

### 3.2 Subsequent-memory imaging analyses

#### 3.2.1 Remembered versus forgotten

##### 3.2.1.1 Group average results

The stimuli were coded according to whether they were subsequently remembered (hits) or forgotten (misses).

**Fig. 2:**
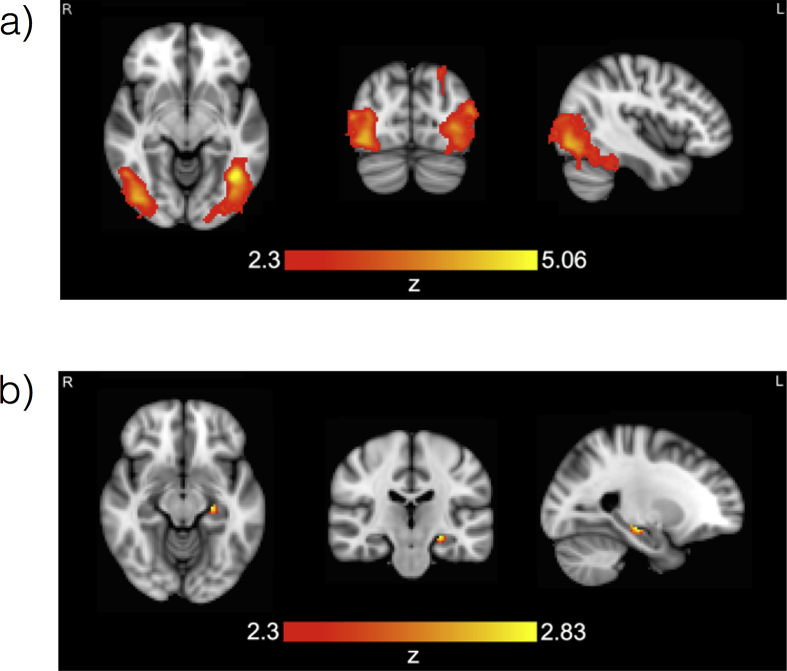
**Group average results for remembered versus** **forgotten.** (a) Whole brain analysis: the brain areas that were significantly more active for subsequently remembered stimuli than for subsequently forgotten stimuli. These are bilateral occipitotemporal regions. They include the lateral occipital cortex, the occipital pole, the occipital fusiform gyrus, the temporo-occipital part of the inferior temporal gyrus and the temporal occipital fusiform cortex bilaterally, in addition to part of the posterior temporal fusiform cortex and the posterior parahippocampal gyrus in the right hemisphere. The maxima can be viewed in Table 3. (b) Left hippocampus region of interest analysis: a region in the left hippocampus that was more active for subsequently remembered stimuli than for subsequently forgotten stimuli. The peak voxel is found at −24, −22, −12 (MNI coordinates, mm), Z = 2.83.

An analysis including all 30 participants was performed to identify brain areas that were more active for subsequently remembered than forgotten stimuli (i.e., that showed a subsequent-memory effect). The results are displayed in Fig. 2a and Table 2.

**Table 2:** Maxima in MNI coordinates (mm) for remembered > forgotten.

Brain region	x	y	z	Z
Inferior temporal gyrus, temporooccipital part	−40	−60	−6	5.06
Lateral occipital cortex, inferior division	−36	−84	4	4.65
	−40	−72	−4	4.33
	−52	−76	14	4.13
Temporal occipital fusiform cortex	−30	−50	−18	3.5
Lateral occipital cortex, superior division	−24	−80	50	3.3
Lateral occipital cortex, inferior division	42	−78	−8	4.32
Occipital pole	32	−92	14	4.09
Lateral occipital cortex, inferior division	50	−80	10	3.99
	42	−68	−14	3.95
Inferior temporal gyrus, temporooccipital part	52	−56	−8	3.79
Lateral occipital cortex, superior division	30	−88	10	3.78

Activations corresponding to subsequent memory occurred in bilateral occipitotemporal regions, and included lateral occipital cortex, occipital pole, occipital fusiform gyrus, the temporo-occipital part of the inferior temporal gyrus and temporal occipital fusiform cortex bilaterally, in addition to part of the posterior temporal fusiform cortex and posterior parahippocampal gyrus in the right hemisphere.

When the analysis was constrained to the left hippocampus, a subsequent-memory effect was also detected in this structure, as displayed in Fig. 2b.

There were no significant results in the right hippocampus.

##### 3.2.1.2 Group difference results

**Fig. 3:**
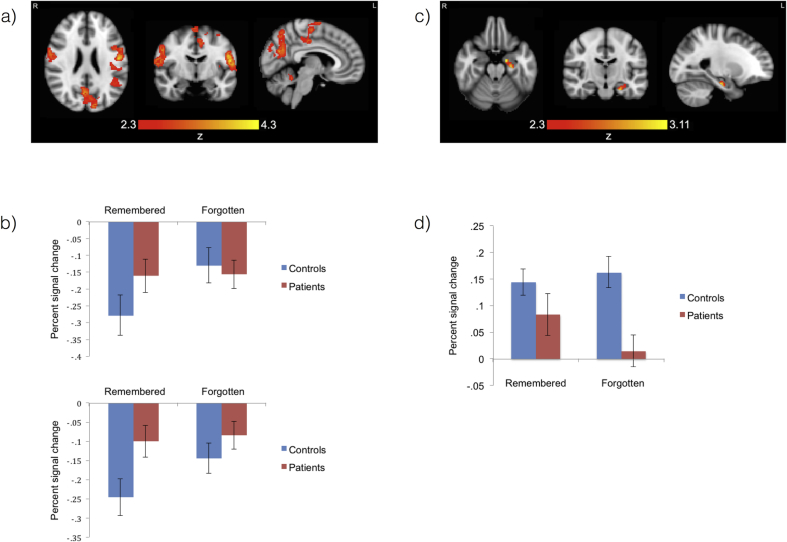
**Patients versus** **controls for remembered versus** **forgotten**. (a) The brain areas in which activation was more positive for subsequently remembered relative to forgotten items in the patients than the controls. These brain areas include regions of the precuneus, the posterior cingulate and the pre- and post-central gyri. The maxima can be viewed in Table 4. (b) Mean percent signal change for the remembered and forgotten contrasts within the precuneus (top) and posterior cingulate (bottom) regions for which activation was more positive for subsequently remembered relative to forgotten items in patients than controls. The controls deactivated these regions for subsequently remembered compared to forgotten items, while the patients did not. The bars each represent a mean across a group of participants. The error bars represent SEMs. c) Left hippocampus region of interest analysis for patients versus controls for remembered versus forgotten: an area in the left hippocampus in which the difference between the activity associated with subsequently remembered and forgotten items was greater in patients than controls. The peak voxel is found at −16, −8, −22 (MNI coordinates, mm), Z = 3.11. (d) A featquery analysis for the whole left hippocampus: the percent signal change associated with subsequently remembered and forgotten items in the left hippocampus. The subsequent-memory effect was significant in the patients only. Percent signal change was significantly lower in the patients than the controls for the subsequently forgotten items only. For controls, both subsequently remembered and forgotten items were associated with left hippocampal activity while, in patients, subsequently remembered items were associated with significant signal change in the left hippocampus while subsequently forgotten items were not.

There were group differences in the subsequent-memory effect. Fig. 3a and Table 3 show brain regions in which the difference between the activation for subsequently remembered and forgotten items was more positive for patients than controls. They include regions of the precuneus, the posterior cingulate and the pre- and post–central gyri.

**Table 3:** Maxima in MNI coordinates (mm) for patients > controls for remembered > forgotten.

Brain region	Z	x	y	z
Postcentral gyrus	4.3	−54	−6	16
Supramarginal Gyrus, posterior division	4.2	−40	−50	18
	4.19	−46	−46	18
Postcentral gyrus	4.04	−52	−10	24
Central opercular cortex	3.86	−56	−10	14
	3.67	−58	−22	18
Precuneus cortex	3.66	6	−68	24
	3.61	2	−64	26
	3.48	2	−64	30
Cuneal cortex	3.48	−10	−88	24
Lateral occipital cortex, superior division	3.43	−14	−86	24
	3.38	−14	−86	20
Postcentral gyrus	3.62	56	−8	36
Central opercular cortex	3.52	56	−14	12
Heschl's Gyrus (includes H1 and H2)	3.52	50	−18	12
Central opercular cortex	3.4	56	−12	18
Precentral gyrus	3.39	56	−6	22
Postcentral gyrus	3.37	60	−6	16
Juxtapositional lobule cortex (formerly supplementary motor cortex)	3.72	2	−2	70
Precentral gyrus	3.67	−4	−18	50
Cingulate gyrus, posterior division	3.43	4	−22	42
	3.38	2	−20	48
Precentral gyrus	3.37	4	−16	60
Juxtapositional lobule cortex (formerly supplementary motor cortex)	3.3	−6	−8	48

The precuneus and posterior cingulate are of particular interest, as they typically deactivate during successful encoding in healthy people, and this deactivation has been shown to fail in populations with memory problems. Therefore, featqueries were performed for the regions of activation that fell within these brain areas. These are plotted in Fig. 3b. In the control group, these regions were deactivated for subsequently remembered compared to forgotten items, but this was not the case in patients.

When the analysis was constrained to the left hippocampus, a cluster of voxels was found for which the subsequent-memory effect was greater in patients than controls. This cluster is displayed in Fig. 3c.

No significant results were found in the right hippocampus.

Subsequently, a featquery analysis was performed for the whole of the left hippocampus. The binary structural mask was transformed into the native space of each participant, where it was used as a mask from which to extract the mean percent signal change associated with the remembered and forgotten lower level contrasts. The results are plotted in Fig. 3d.

An ANCOVA with percent signal change as the dependent variable, subsequent memory as the within-subjects factor (two levels: remembered and forgotten), group as the between subjects factor and depression score on the HAD scale as a nuisance covariate, demonstrated an interaction between subsequent memory and group [F_(1,27)_ = 7.01, *p* = .013]. The subsequent-memory effect was significant in patients only (*p* = .002), and not controls (*p* = .58). The percent signal change in the left hippocampus was significantly lower in patients than controls for the subsequently forgotten items only (*p* = .018), and not the remembered items (*p* = .86).

In the controls, the percent signal change was significantly greater than zero for both the subsequently remembered [t_(14)_ = 5.92, *p* < .001] and forgotten items [t_(14)_ = 5.55, *p* < .001]. However, in patients, the percent signal change was significantly greater than zero for subsequently remembered items [t_(14)_ = 2.42 *p* = .030], but not for subsequently forgotten items [t_(14)_ = .50, *p* = .63].

A whole brain analysis revealed no brain areas in which controls had a larger subsequent-memory effect than the patients.

##### 3.2.1.3 Subsequent-memory effects for the late test versus the early test

**Fig. 4:**
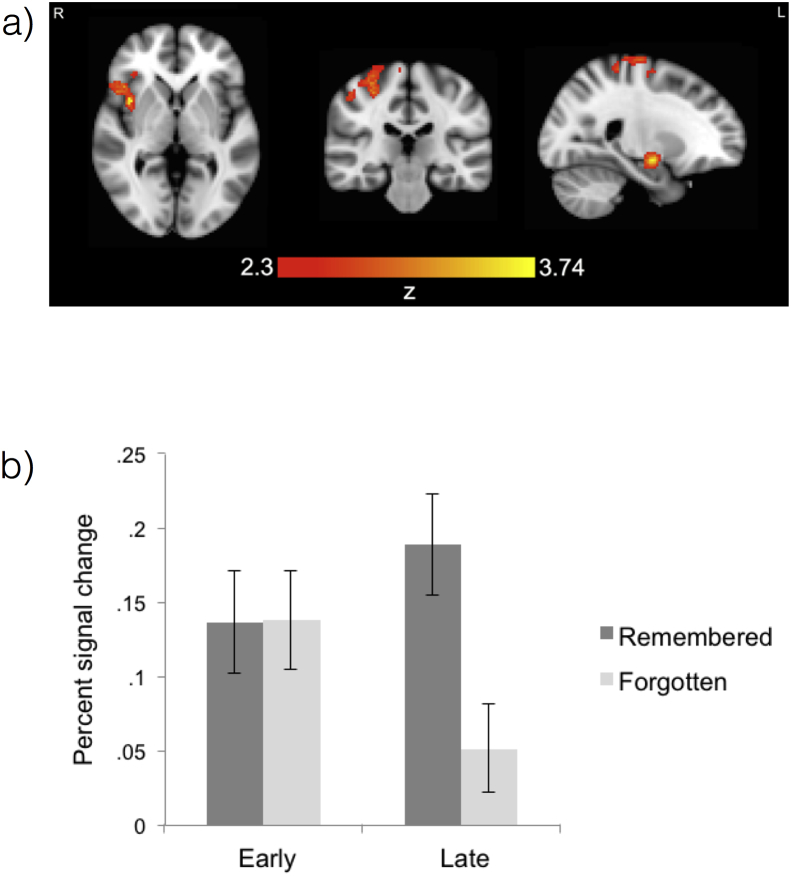
**Late versus** **early subsequent-memory effects.** (a) The brain areas in which the subsequent-memory effect was significantly greater for the Late test than the Early test. The areas include the right post- and pre-central gyri, the right insular cortex and the right amygdala. The maxima can be viewed in Table 4. (b) Mean percent signal change for the remembered and forgotten contrasts within the area of the right amygdala that showed a significantly greater subsequent-memory effect for the Late test than the Early test.

In a whole brain analysis across both groups, a number of brain regions exhibited a larger subsequent-memory effect for Late test items than for Early test items. These are displayed in Fig. 4a and Table 4 and include the right post- and pre-central gyri, the right insular cortex and the right amygdala.

**Table 4:** Maxima in MNI coordinates (mm) for Late remembered > Late forgotten > Early remembered > Early forgotten.

Brain region	x	y	z	Z
Insular cortex	40	10	0	3.74
Amygdala	24	−8	−12	3.68
Insular cortex	38	14	−6	3.5
	34	18	−8	3.47
	38	8	−6	3.37
Frontal operculum cortex	46	20	2	3.28
Precentral gyrus	30	−26	52	3.15
	32	−26	60	3.12
Superior frontal gyrus	28	−6	62	3.09
Postcentral gyrus	30	−28	64	3.03
Precentral gyrus	−6	−16	68	3.03
Postcentral gyrs	40	−38	66	3.03

As an MTL region, the amygdala result is of particular interest. Therefore, for illustrative purposes, a featquery for the region of activation that fell within the amygdala is shown in Fig. 4b.

There were no brain areas in which there was a larger subsequent-memory effect for the Early test than the Late test.

There were no significant group differences for either of these contrasts.

## 4 Discussion

This is the first study to investigate brain activity during memory encoding in patients with TEA. The key finding is that these patients, who perform normally on standard anterograde memory tests but complain of, and demonstrate, accelerated forgetting thereafter, have abnormal brain activity at the stage of encoding that predicts subsequent memory performance. In addition, across the whole participant group, encoding activity in limbic regions including the right amygdala predicted the longevity of memory. In line with previous studies, we found that memory impairment emerged over different time intervals on different tasks, a result that has important implications for the development of clinical measures of forgetting. We discuss these findings in turn.

### 4.1 Patients with TEA show encoding-related abnormalities in brain activity

The patients showed a different subsequent-memory effect from controls in memory-critical brain areas including the left hippocampus (in an ROI analysis), precuneus and posterior cingulate.

The group difference in the subsequent-memory effect in the hippocampus was driven by hypoactivity associated with subsequently forgotten stimuli in the patients. This hippocampal hypoactivity may indicate the formation of substandard memory representations that do not support subsequent retrieval. One interesting possibility is that such poorly formed memory representations may be more vulnerable to interference from similar information (Ally et al., 2013, Yassa and Stark, 2011). Indeed, patients with MTL lesions have been shown to be especially sensitive to interference (e.g., Cowan et al., 2004, Dewar et al., 2009, Warrington and Weiskrantz, 1978). Similarly, we previously found patients with TEA to show a memory benefit under conditions of reduced interference (sleep *vs* wake) and only to show accelerated forgetting in the interference (wake) condition (Atherton et al., 2014).

The fact that patients with accelerated forgetting display abnormal brain activity at the stage of encoding does not mean that they do not also experience problems related to memory consolidation. Substandard encoding may render memory traces particularly vulnerable to interference from similar information, with the result that the reactivation of related older memories during systems consolidation (e.g., McClelland, McNaughton, & O'Reilly, 1995) could promote catastrophic interference. Indeed, slow wave sleep, which is associated with systems consolidation, appears to be deleterious for memory in TEA patients with accelerated forgetting (Atherton et al., 2016). To test for enhanced susceptibility to interference in patients with accelerated forgetting, the degree of feature overlap between the information to be encoded, retroactive interference in the retention interval, and the foils could be experimentally manipulated.

It is notable that we found no difference in hippocampal encoding activity between subsequently remembered and forgotten stimuli in the control group. This is not uncommon in subsequent memory experiments. Several factors have been proposed that may affect the degree to which hippocampal subsequent memory effects are found (Henson, 2005, Kim, 2011) including i) signal dropout from the MTL, ii) whether or not confidence measures are taken into account, with studies that look at high confidence responses only showing greater sensitivity and iii) the nature of the stimulus material, with associative memory tasks showing greater subsequent memory effects than item memory tasks. In our task, signal dropout would not appear to be a satisfactory explanation since robust signal change was observed in the hippocampus for both remembered and forgotten stimuli, and a clear subsequent memory effect was shown in patients. However, our task probed item recognition memory only and our ‘remembered’ items did not only include high confidence responses, so these factors may have played a role. It is possible that a certain degree of input from the hippocampus is necessary to produce a representation that can be adequately disambiguated from others but that, once this criterion is met, additional hippocampal activity has little impact and other factors determine memory performance.

The patients also demonstrated reduced deactivation of posteromedial cortex regions for stimuli that were subsequently remembered. Interestingly, this has previously been observed in older adults with memory impairments (Miller et al., 2008), those with mild cognitive impairment (Celone et al., 2006), and those with Alzheimer's disease (Pihlajamaki et al., 2008, Pihlajamaki et al., 2010). Posteromedial cortex is known to be a critical part of a hippocampus-dependent memory network (Buckner et al., 2008, Miller and D'Esposito, 2012, Sperling et al., 2010, Spreng et al., 2009, Wang et al., 2010). Its precise role in memory encoding is unknown, but the decrease in BOLD signal associated with encoding has been proposed to reflect the allocation of attention to external stimuli (Huijbers et al., 2012). Other regions showing group differences in this contrast, such as pre- and post–central gyri, are commonly identified in subsequent memory experiments (Kim, 2011) and may reflect differences in attentional regulation during encoding or distinct motor responses to subsequently remembered versus subsequently forgotten stimuli.

Our results are consistent with the notion that accelerated forgetting in TEA is the result of a functionally compromised hippocampal memory system. This notion gains support from the finding that the rate of forgetting in epilepsy patients depends on the degree of hippocampal pathology; patients with hippocampal lesions tend to exhibit memory problems shortly after encoding, while those without hippocampal lesions (whose functional impairment is presumably milder) often only show memory deficits after longer retention intervals (Lah et al., 2014, Wilkinson et al., 2012). Strikingly, recent evidence shows that pre-symptomatic individuals at genetic risk of both familial (Weston et al., 2018) and sporadic (Zimmermann & Butler, 2018) Alzheimer's disease suffer from forgetting over the longer term, but not yet over the shorter term. This suggests that subtle MTL dysfunction causes longer-term forgetting and that, as the MTL degenerates, memory impairments manifest progressively earlier.

### 4.2 Encoding-related activity differences predict memory longevity across whole cohort

Our whole-brain analysis across all participants showed that subsequently remembered stimuli were associated with more activity in occipitotemporal regions, including the fusiform cortex and posterior parahippocampal gyrus, than subsequently forgotten stimuli. This result is consistent with many previous studies (e.g., Garoff et al., 2005, Kirchhoff et al., 2000, Wagner et al., 1998). However, no significant clusters were found in other commonly identified areas such as prefrontal or dorsal posterior parietal cortex. This may be a consequence of shallow stimulus processing, since the encoding task required minimal elaboration. Within a hippocampal ROI we detected a cluster of left hippocampal voxels in which there was greater activity in response to subsequently remembered than forgotten items.

Across all participants, a whole-brain analysis revealed various regions in which there was a greater subsequent-memory effect for Late test items than for Early test items. These included a large cluster covering the right amygdala, along with regions of the insular cortex and the pre- and post–central gyri. The influence of retention interval on the subsequent-memory effect suggests that successful recognition at a late time point requires neural processing at the stage of encoding over and above that required for successful recognition shortly after learning. The involvement of the amygdala is interesting. A large body of research indicates that emotional arousal enhances the subsequent retention of long-term declarative memory and that this effect is mediated by the amygdala (e.g., Cahill and McGaugh, 1998). Our results suggest that amygdala involvement at the stage of encoding results in durable memories. However, it should be noted that this region of activation was close to the right anterior hippocampus and so, given that the analyses involved registration and smoothing, it is possible that some of this activation was actually hippocampal. We also identified a larger subsequent memory effect for Late versus Early test items in the right insula. This region is a core part of the salience network, suggesting that its involvement in encoding enduring memories may reflect greater attentional capture by certain stimuli at presentation.

### 4.3 Time course of memory deficits is task-dependent

Our finding that patients with TEA showed accelerated forgetting over the longer term (i.e., ALF) on a test of verbal recall but earlier deficits on a visual recognition task is consistent with other recent studies. Dewar, Hoefeijzers, Zeman, Butler, & Sala, Della (2015) found picture recognition memory impairments five minutes after learning in a group of TEA patients, while a much longer retention interval was required before deficits were detectable on a verbal memory task. A detailed case report of a patient with ALF due to baclofen treatment revealed a very similar pattern of results (Zeman et al., 2016). Similarly, in temporal lobe epilepsy patients, Cassel, Morris, Koutroumanidis, & Kopelman (2016) only found statistically significant memory impairment on a story recall task a week after learning, while the same patients demonstrated memory problems within 10 min on a visuospatial task, and no accelerated forgetting thereafter.

It is possible that systematic differences in the course of accelerated forgetting arise from the nature of the memoranda (verbal *vs* visuospatial) or task process demands (recall *vs* recognition memory), and this should be examined in future studies. A further potential explanation relates to the idea that forgetting occurs due to mutual interference between similar memoranda. The reduced d’ seen in our patient group on the Early recognition memory test was due to an increased false alarm rather than decreased hit rate. This pattern is similar to that identified by Dewar et al. (2015) in patients with TEA and is consistent with memory traces being less well specified and thus more susceptible to interference. The time at which such interference occurs will determine the time at which forgetting becomes apparent. In a word recall task, there are relatively few, distinct stimuli at encoding (15 unrelated words in this case), so interference might be expected to arise only from an extended retention interval during which other words were encountered. In contrast, if a task involves the encoding of a large number of novel stimuli that have many overlapping features (as was the case in our experimental task), these stimuli may interfere with each other during the encoding period. The correlation that we identified between performance on our experimental task and 1-week forgetting on the word recall task provides some behavioural support for similar underlying forgetting mechanisms across tasks, although this will need to be examined systematically in future research.

The clinical importance of ALF amongst neurological patients is increasingly recognised. Novel neuropsychological tests, sensitive and specific to longer-term forgetting, are required. In developing such tests, it will be critical to understand the way in which different task parameters affect forgetting rates in health and disease.

### 4.4 Limitations

The sample size in this study was relatively small. TEA is a moderately rare condition. It is, nevertheless, an ideal population in which to investigate accelerated forgetting since this memory deficit is generally found in relative isolation compared with the wider cognitive problems seen in other forms of temporal lobe epilepsy (Bell, Lin, Seidenberg, & Hermann, 2011). Similar sample sizes have been used in previous studies using fMRI to investigate the neural mechanisms of cognitive dysfunction in epilepsy (e.g., Glickmann-Johnston et al., 2008).

Studies of ALF have traditionally used verbal and/or visual recall tasks (Elliott, Isaac, & Muhlert, 2014). Our choice of a recognition memory task rather than a recall task for this study was guided by the need to have a large number of stimuli for the subsequent memory analysis. As a consequence, we cannot be certain that our results generalise to these other tasks even though our patients did show the standard pattern of ALF on the RAVLT word list task. Moreover, the design of our fMRI experiment prevented us from examining very early memory retention (e.g., after 40 sec) in the way that we could with the RAVLT. Future fMRI encoding experiments in accelerated forgetting should employ verbal or visual recall tasks in which the memory deficit can be shown to manifest only at later time points.

## 5 Conclusions

In conclusion, we have detected abnormal encoding-related brain activity among patients with TEA who show normal learning but accelerated forgetting on standard memory tasks. Notably, patients exhibited hypoactivity in the left hippocampus for items they would go on to forget, together with differential activity levels in other parts of the wider memory network. This abnormal encoding activity may reflect the formation of substandard memory representations that are vulnerable to interference. Future studies should examine whether these findings can be extended to other conditions associated with accelerated forgetting such as preclinical Alzheimer's disease. They should also test the hypothesis that patients with accelerated forgetting are especially vulnerable to interference and investigate whether this vulnerability is specific to stimuli associated with hippocampal hypoactivity at encoding.

## Funding

K.E.A. and A.C.N. (Senior Investigator Award 104571/Z/14/Z) were supported by the Wellcome Trust. C.R.B. holds a Medical Research Council Clinician Scientist fellowship (MR/K010395/1). This research was supported by the NIHR Oxford Health Biomedical Research Centre. The Wellcome Centre for Integrative Neuroimaging is supported by core funding from the Wellcome Trust (203139/Z/16/Z).
